# Variation in SSRs at different genomic regions and implications for the evolution and identification of *Armillaria gallica*

**DOI:** 10.1371/journal.pone.0312114

**Published:** 2024-10-15

**Authors:** Shengnan Li, Jiao Xu, Yipu Hu, Xiaohong Ou, Qingsong Yuan, Pengfei Li, Weike Jiang, Lanping Guo, Tao Zhou

**Affiliations:** 1 Guizhou University of Traditional Chinese Medicine, Guiyang, China; 2 National Resource Center for Chinese Materia Medica, State Key Laboratory Breeding Base of Dao-di Herbs, China Academy of Chinese Medical Sciences, Beijing, China; Government College University Faisalabad, PAKISTAN

## Abstract

*Armillaria* spp. are devastating forest pathogens. Due to its low pathogenicity and abundant genetic variation, *Armillaria gallica* exhibited a unique and beneficial symbiosis with *Gastrodia elata*, which was used as a traditional Chinese medicine. However, the variation and population structure of *A*. *gallica* populations have rarely been investigated. Hence, we analyzed the evolution and variation in simple sequence repeats (SSRs) in three Armillaria genomes: *A*. *gallica*, *A*. *cepistipes*, and *A*. *ostoyae* to assess the genetic diversity and population structure of 14 *A*. *gallica* strains. Genome analysis revealed that SSRs were more abundant in the intergenic region than the intron and exon region, as was the SSR density. Compared with other two genomes, SSR density was the lowest in exon region and largest in the intron region of *A*. *gallica*, with significant variation in genic region. There were 17 polymorphic markers in *A*. *gallica* genome was identified, with 26.7% in genic region, which is higher than that of 18.8% in the intergenic region. Moreover, a total of 50 alleles and 42 polymorphic loci were detected among these *A*. *gallica* strains. The averaged polymorphism information content (PIC) was 0.4487, ranged from 0.2577 to 0.6786. Both principal coordinate analysis (PCoA) and population structure analyses based on the genotype data of SSRs divided the strains into two clusters. The cluster I included all the strains from high-altitude *G*. *elata* producing areas and some low-altitude areas, while the strains in Cluster II originated from low-altitude *G*. *elata* producing areas. These results indicated that substantial genome-specific variation in SSRs within the genic region of *A*. *gallica* and provide new insights for further studies on the evolution and breeding of *A*. *gallica*.

## 1. Introduction

Armillaria is a facultative parasitic fungi with approximately 52 reported species [1,2], of which about 15 species have been identified in China [3]. Most Armillaria species are devastating forest pathogens that cause root rot disease in forests. Only a few of them with low pathogenicity such as *Armillaria gallica* can form a symbiotic relationship with *Gastrodia elata*, a mycoheterotrophy plant in the orchid family benefiting from *A*. *gallica* [4–9] and a special symbiotic relationship is established [10]. The dried tuber of *G*. *elata* is commonly used as a traditional Chinese medicine with high medicinal value in anticonvulsant, memory improving, and other neurological pharmacological effects [11–15].

*A*. *gallica* is commonly used as the companion strain for *G*. *elata* cultivation. The growth and development of *G*. *elata* vary significantly depending on the symbiotic relationship established with different varieties of *A*. *gallica* [16]. The cultivation areas of *G*. *elata* spans diverse climatic regions in China, such as the Hubei, Guizhou and Tibet, resulting in a broad genetic diversity of *A*. *gallica* strains across the different regions [17]. In addition, *A*. *gallica* from different habitats was introduced frequently, which can easily lead to homonymy (same name for genetically different strains) or synonymy (same strains with different names) of *A*. *gallica*. The efficient identification of *A*. *gallica* strains is therefore important.

Investigating the population genetics of *A*. *gallica* is complex due to its varied mating systems, including sexual reproduction, clonal (asexual) spread, and diploid–haploid mating [18,19]. The effective identification of *A*. *tobescens*, *A*. *ostoyae*, and *A*. *mellea* was established using intergenic spacer-restriction fragment length polymorphism (IGS-RFLP) [20]. In addition, the ribosomal DNA-internal transcribed spacer (rDNA-ITS) has been used to identify *A*. *mellea*, *A*. *gallica*, and *A*. *cepistipes* [21]. PCR-RFLP identified two types of *A*. *gallica* with low or high sugar contents [22]. However, the heredity and variation among populations of *A*. *gallica* have not been elucidated, and there is still a lack of suitable methods for the identification of *A*. *gallica*.

Simple sequence repeats (SSRs) are DNA segments with repeat motifs of one to six nucleotides. SSRs are widely used in assessments of the genetic diversity of germplasm resources, the construction of population genetic maps, and molecular marker-assisted breeding [23]. SSR polymorphism or variation may affect the evolution of the genome under control of evolutionary forces [24]. Furthermore, impaired DNA mismatch repair was one of the reasons for high mutation rate of SSR, which the polymorphism or variation of SSR may affect genome evolution [25]. SSRs can be categorized into genomic SSRs (gSSR) and expressed sequence tag SSRs (EST-SSR), each derived from genomic DNA sequence and expressed sequence tags, respectively [26]. gSSR generally exhibit higher polymorphism than EST-SSR. The PCR amplified fragments were not affected by introns in the flanking region. SSR variation in intronic SSRs could affect gene transcription, or mRNA splicing. SSRs located in protein-coding regions could lead to functional gene variation via frameshift mutation, contributing to rapid evolutionary changes [27]. Studying SSR patterns across different species might provide insight into genome evolution [28]. Large number of SSRs have been developed for the identification of plants or microorganisms [29,30]. However, the available number of SSR markers **is** relative limited in *Armillaria spp* [31,32], with even fewer reported for *A*. *gallica*. With the development of genome and transcriptome sequencing have enhanced the efficiency of SSR development, contributing greatly to the identification of Chinese medicinal materials.

Hence, it is particularly important to study the evolution and identification research of *A*. *gallica*. In this study, genome data for three Armillaria species were used to analyze the distribution of SSRs in the both genic and intergenic region. The patterns and variations of SSRs in *A*. *gallica* were examined. We also collected *A*. *gallica* strains from various *G*. *elata* production areas and assessed the genetic diversity. We hope our findings would shed light on genome evolution, genetic research and identification of *A*. *gallica*.

## 2. Materials and methods

### 2.1 Armillaria sources

Three previously published Armillaria genomes including *A*. *gallica* (VTST01000000), *A*. *cepistipes* (FTRY01000001-FTRY01000182), and *A*. *ostoyae* (FUEG01000001-FUEG01000106), were downloaded in this study [33,34]. The three genomes, *A*. *gallica*, *A*. *cepistipes* and *A*. *ostoyae*, had similar genetic background, and the expansion of protein-coding gene pool in Armillaria was similar. *A*. *ostoyae* was hard to symbiosis with *G*. *elata* due to its strong pathogenicity, *A*. *gallica* and *A*. *cepistipes* could symbiosis with *G*. *elata*, but *A*. *gallica* had the best affinity [7,33,35]. The strains of *A*. *gallica* that are symbiotic with *G*. *elata* were collected from Guizhou, Hubei, Shanxi, Yunnan, and Henan provinces as well as from other *G*. *elata* cultivation areas or research institutions. The detailed information of these strains are listed in S1 Table. Three Armillaria genomes were used to analyze the features of SSR motifs.

### 2.2 IGS identification

An Ezup column fungal genomic DNA extraction kit (Shanghai Sangong Bioengineering Co., Ltd.) was used to extract the total genomic DNA. The rDNA-IGS sequences were amplified and compared with the Armillaria species in GenBank for the interspecific identification of Armillaria [36].

### 2.3 Development and identification of SSRs

MISA software was used to analyze the SSRs in the reference genome of *A*. *gallica*. (http://misaweb.ipk-gatersleben.de/). Parameters were set based on previous study [24,37]. The minimum repeat unit is set to mononucleotide motif containing 10 or more repeats, dinucleotide motif containing 6 or more base repeats, trinucleotide, tetranucleotide, pentanucleotide, hexanucleotide and complex motif containing 5 or more base repeats. When the distance between two SSRs was less than 100 bp, the two SSRs formed a complex motif. The data of each SSR motif were extracted, and the number of mononucleotides, dinucleotides, trinucleotides, tetranucleotides, pentanucleotides, hexanucleotides, complex nucleotides, and multi-complex nucleotides, totaling eight types of SSR motifs, were counted and analyzed to characterize the SSR motifs on the genomes of the three Armillaria.

### 2.4 SSR distribution in different genomic regions

The genic region (intron, exon) and intergenic region were extracted from GFF files of each Armillaria genome. The genic region (intron, exon) and intergenic region were extracted from GFF files of each Armillaria genome by TBtools. According to the previous study, the quantity and length of SSRs in the different genomic regions were developed to identify [24,38]. And 108 pairs of primers were randomly selected including eight SSR motifs during genic region and intergenic region.

### 2.5 PCR amplification and genotyping

Primer 3.0 software was used to design flanking primers with the sequences following the detected putative SSRs. The SSR primer pairs were randomly selected during all scaffolds to assess the quantity of the developed SSRs. The SSR primers were synthesized at Sangong Bioengineering (Shanghai) Co., Ltd. The PCR was performed in 10 μL reaction volume containing10×Easy Taq Buffer (10 mM Tris-HCl pH 9.0, 1.5 mM MgCl_2_, 50 mM KCl), 0.1 mM High Pure dNTPs, 0.1U Taq DNA polymerase, 0.1 mM each of forward and reverse primers, and 50 ng of genomic DNA was used as template in each PCR reaction. PCR program was as follows: initial denaturation at 95°C for 5 min, These cycles were subsequently followed by 32 cycles of denaturation at 95°C for 30 s with a constant annealing temperature of 58°C for 30 s, and extension at 72°C for 45 s, and a final extension of 72°C for 5 min. The amplified products were separated by 8% non-denatured polyacrylamide gel electrophoresis, stained with 0.1% silver nitrate solution, developed in the developer solution, and photographed for preservation.

### 2.6 SSR data analysis

The same band type amplified by each pair of SSR primers on the test material was considered an allelic variation, and according to the relative position of the separated bands, those with bands were marked as 1, while those without bands were marked as 0, forming a 0/1 matrix [39].

NTSYS 12.0 software was used to calculate the genetic correlation coefficient and genetic distance was used to cluster the samples based on the unweighted pair-group method with arithmetic means (UPGMA), and the neighbor joining tree was constructed [40]. Popgene32 software was used to calculate the average number of observed alleles (Na), the number of effective alleles (Ne), allele Frequency and the Shannon diversity index (Nei’s genetic identity, I) [41]. The polymorphism information content (PIC) was equal to 1 minus the sum of the squared frequencies of all alleles [42]. The principal coordinates analysis (PCoA) map was obtained based on GenAIEx 6.5 software [43].

The population structure analysis was performed by the Bayesian model-based clustering method in Structure v.2.3.4. The number of populations (K) was from 1 to 10, and each K value was iteratively calculated 10 times. The MCMC value and burn-in value were set to 150,000 and 10,000, respectively [30,44]. The optimal K value was determined by ΔK methodology using the web-based software StructureSelector [45].

## 3. Results

### 3.1 Armillaria genomic sequences

**Fig 1:**
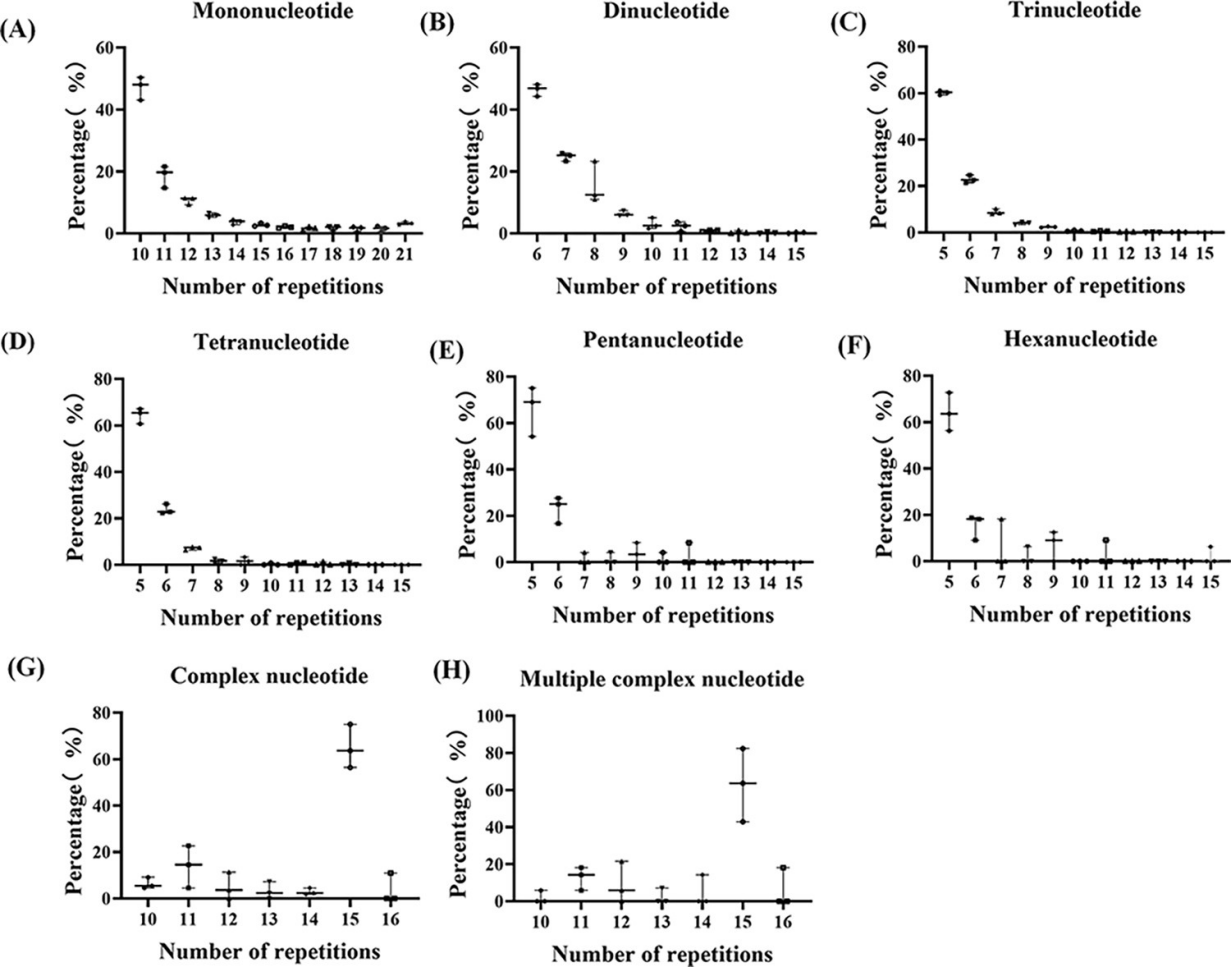
Length polymorphism of eight the nucleotide motifs. (A) Mononucleotide, (B) Dinucleotide, (C) Trinucleotide, (D) Tetranucleotide, (E) Pentanucleotide, (F) Hexanucleotide, (G) Complex nucleotide, (H) Multiple complex nucleotide.

The number of SSRs in genomic was identified in *A*. *gallica* (4,141) which was much higher when compared with *A*. *cepistipes* (3,116), and *A*. *ostoyae* (2,336). The data indicated that *A*. *gallica* which had the largest genome size contains the highest number of SSRs. Total relative abundance and total relative density was calculated to estimate the SSRs by taking 1 Mb length of each set of sequences. *A*. *cepistipes* (41.88 and 647.42) had the maximum frequency of SSRs, while *A*. *gallica* had the similar frequency as *A*. *ostoyae* (Table 1). Different types of SSR motifs in the three genomes were further analyzed. Finally, expecting the complex and multiple complex type nucleotide, the frequency of repeat motifs decreased as the number of repeat motifs increased (Fig 1A–1F, S2 Table). In addition, in the mononucleotide to hexanucleotide motif, the first two base repeats accounted for the highest proportion, and were the most common in *Armillaria* genomes and account for more than 60% of the total repeats (Fig 1, S2 Table).

**Table 1:** Armillaria genome SSR size, length, No. of SSR, relative abundance and relative density.

	*A*. *cepistipes*	*A*. *gallica*	*A*.*ostoyae*
Size (Mb)	74.4	106.8	60.1
No. of SSR	3116	4141	2336
Perfect SSR	3058	4075	2297
%	98.14	98.41	98.33
Compound SSR	44	55	22
%	1.41	1.33	0.94
SSRs length (bp)	48168	62667	35186
abundance (SSR/Mb)	41.88	38.77	38.87
density (bp/Mb)	647.42	586.77	585.46

We further analyzed the percentage of different classes of repeats in their respective genomes. The mono-, di-, and trinucleotide motifs exhibited at least 10 times as abundant as other five motifs (Table 2). We further analyzed the percentage of different classes of repeats in their respective genomes. In A. *cepistipes*, *A*. *gallica*, and *A*.*ostoyae* mononucleotide repeats constituted the maximum percentage of SSRs (43.58, 43.25 and 39.51%) followed by trinucleotide repeats (29.85, 31.54 and 32.62%) while hexanucleotide repeats were the least (0.51, 0.27 and 0.47%; Table 2).The average of quantity variation was 0.28% between *Armillaria* genomes, with genome variation being larger in penta- (0.69%) and complex nucleotides (0.42%) motifs.

**Table 2:** The quantity of eight SSR motifs in three Armillaria strains genomes.

	Mononucleotide			Dinucleotide			Trinucleotide		
Species	Count	Percentage(%)	CV(%)	Count	Percentage(%)	CV(%)	Count	Percentage(%)	CV(%)
*A*. *cepistipes*	1358	43.58	0.32	603	19.35	0.23	930	29.85	0.28
*A*. *gallica*	1791	43.25		812	19.61		1306	31.54	
*A*.*ostoyae*	923	39.51		518	22.17		762	32.62	
	Tetranucleotide			Pentanucleotide			Hexanucleotide		
Species	Count	Percentage(%)	CV(%)	Count	Percentage(%)	CV(%)	Count	Percentage(%)	CV(%)
*A*. *cepistipes*	122	3.92	0.25	29	0.93	0.69	16	0.51	0.23
*A*. *gallica*	130	3.14		25	0.60		11	0.27	
*A*.*ostoyae*	79	3.38		4	0.17		11	0.47	
	Complex nucleotide			multiple complex nucleotide					
Species	Count	Percentage(%)	CV(%)	Count	Percentage(%)	CV(%)			
*A*. *cepistipes*	44	1.41	0.42	14	0.45	0.21			
*A*. *gallica*	55	1.33		11	0.27				
*A*.*ostoyae*	22	0.94		17	0.73				

### 3.2 Comparative analysis of the number and density of different SSR motifs in different regions of Armillaria genome

In order to analyze the number of SSRs distributed in different regions in the genomes of the three Armillaria, the distribution of SSRs in the intergenic region and the genetic region (exon and intron region) of the Armillaria were compared. The results showed that the quantity and density of SSRs in intergenic region was more than that in genic region. Among the three genomes, the number of SSRs was the highest in the intergenic region, followed by exons and introns. However, SSR density was the lowest in exon region and highest in the intron region of *A*. *gallica* compared with other two genomes (Table 3). The results showed that genic region had large genome-specific variation in *A*. *gallica* genome.

**Table 3:** The size of Armillaria genome, SSRs length, No. of SSR and SSR relative in genomic regions.

	region	*A*. *cepistipes*	*A*. *gallica*	*A*.*ostoyae*
Size (Mbp)		74.37	106.82	60.08
SSRs length (bp)	intergenic	32317	39555	21227
	intron	5194	12722	3794
	exon	10657	10390	10165
	genetic region	15851	23112	13959
No. of SSR	intergenic	2134	2651	1458
	intron	380	847	310
	exon	602	643	568
	genetic region	982	1490	878
density (bp/Mbp)	intergenic	434.57	370.28	353.33
	intron	69.84	119.09	63.15
	exon	143.30	97.26	169.20
	genetic region	213.15	216.35	232.35

**Fig 2:**
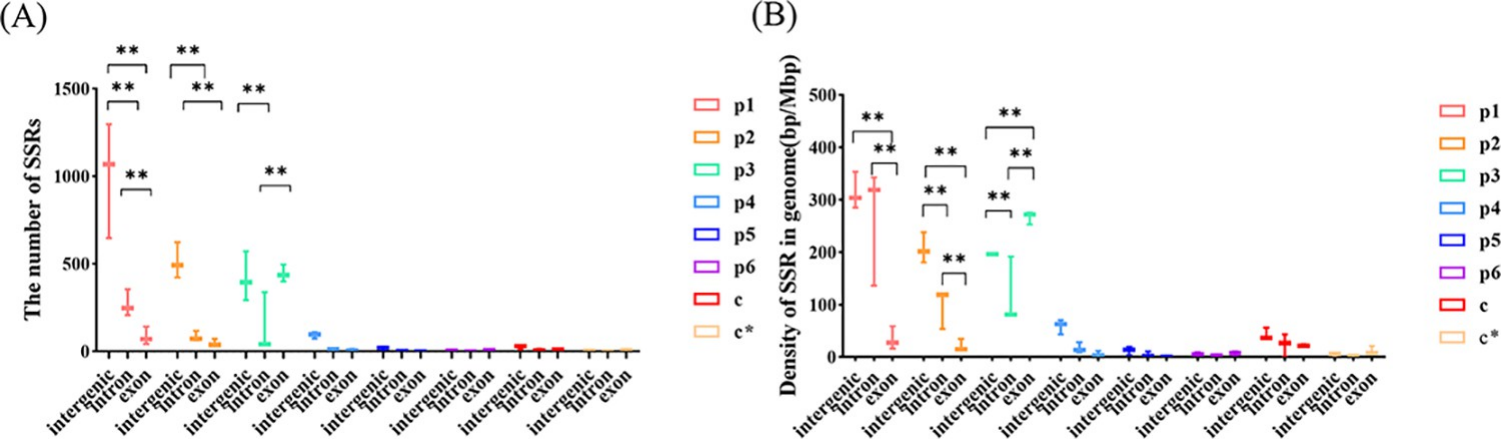
The Quantity (A) and Density (B) of eight SSR motifs in three genome regions (**P<0.01).

To analyze the number and density of different SSR motifs in the three genomes, SSR motif in different genic region was compared and analyzed. For all eight SSR motifs, the difference of SSR quantity and density were mainly in mono-, di-, and trinucleotide motifs among the three regions. For mono- and dinucleotide motifs, the largest and the least SSR quantity and density were distributed in intergenic region and exon region, respectively. However, the trinucleotide motifs mainly distributed in the exon region (Fig 2A and 2B). The results revealed the variation of SSRs with trinucleotide motif was the main reason for large variation in genic region of *A*. *gallica*.

### 3.3 DNA polymorphism

**Fig 3:**
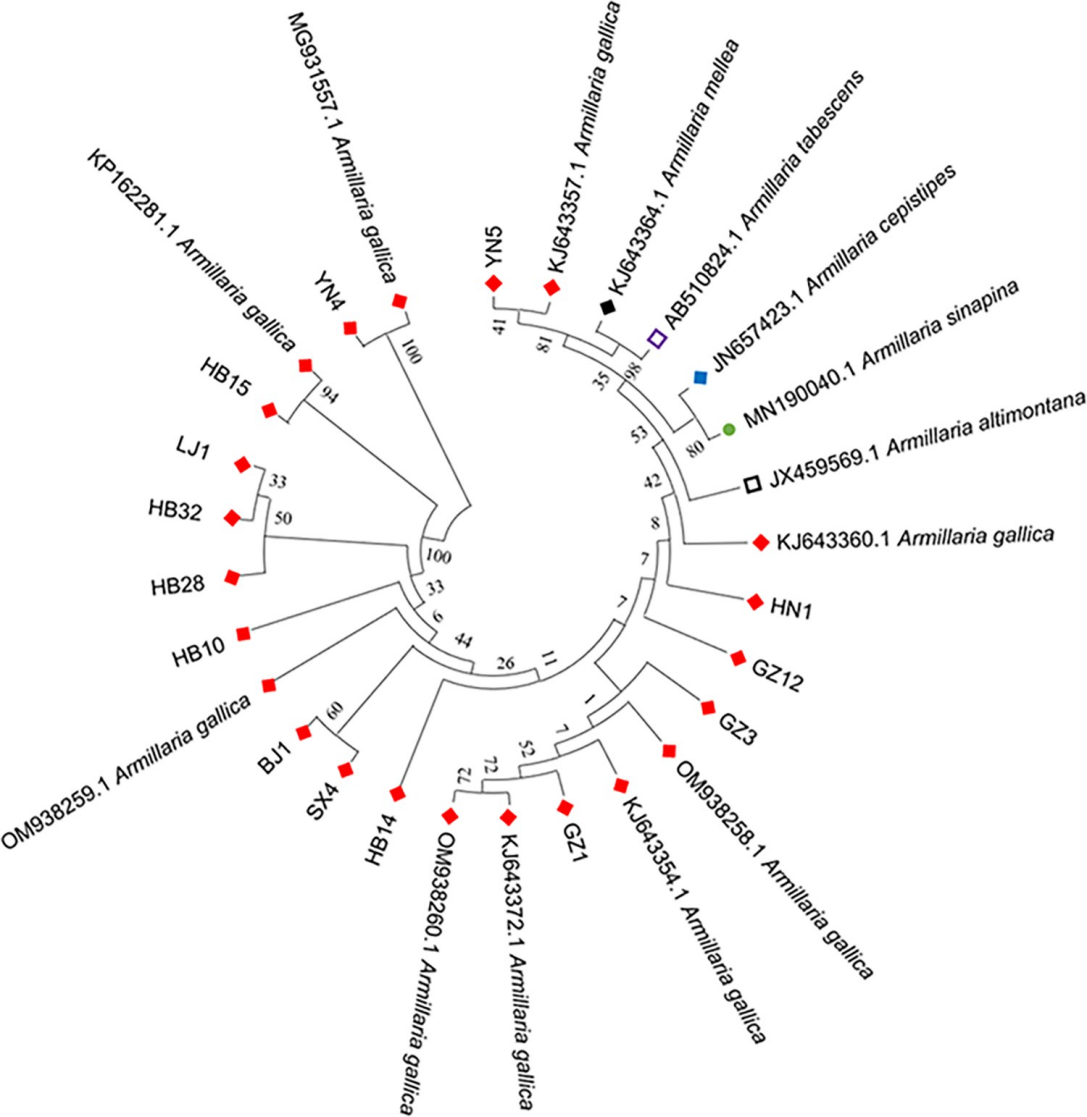
Phylogenetic analyses based on IGS sequences in the Armillaria strains.

Fourteen strains of Armillaria were collected from various *G*. *elata* cultivation areas in China. And phylogenetic analysis showed that all the strains had the highest similarity and the closest genetic relationship with *A*. *gallica*. This result indicated that the 14 strains belong to *A*. *gallica* IGS analysis was used for interspecific identification (Fig 3).

The genome of A. *gallica* contained different types of SSRs, from mononucleotide to hexanucleotide repeat types. Approximate 108 SSR markers s were randomly selected in the *A*. *gallica* genome for genotyping. According to the location in the genome, it was found that 63 SSR markers were in the intergenic region, followed by 45 SSR markers in genic region. They were accounted for 58.3%, 41.7% in total 108 markers, respectively (S3 Table). The results showed that 17 SSR markers were polymorphic (S4 Table), with 12 markers distributed in genic region, and 5 markers in intergenic region. The proportion of polymorphism markers amplified by genic region was 26.7%, which was higher than that of the intergenic region 18.8%. A total of 50 polymorphic loci were observed, of which 42 belong to 9 SSR markers with alleles ranged from two to five, with 42 polymorphic loci and a polymorphism ratio of 84%. The results showed that the proportion of polymorphic SSR markers in genic region was higher than that in intergenic region.

### 3.4 Genetic diversity analysis of *A*. *gallica*

These 17 SSR markers were used to investigate genetic diversity and population structure in 14 strains of *A*. *gallica*. The genetic correlation coefficient was between 0.4400 and 1.000 (S5 Table), and the genetic distance (D) was between 0 and 0.8614.The YN4 and BJ1 exhibited the farthest genetic distance of 0.8614. In addition, the average number of observed alleles (Na) was 3, and the average number of effective alleles (Ne) was 1.9149. The average Nei’s diversity index (H) and the average Shannon index (I) were 0.4487and0.7587, respectively. Furthermore, the observed heterozygosity (Obs_Het) ranged from 0 to 0.7143 with an average of 0.3866, and the expected heterozygosity (Exp_Het) ranged from 0.2672to 0.7037 with an average of 0.4653. The expected heterozygosity is lower than the observed heterozygosity, indicating excessive heterozygosity. Polymorphism information content (PIC) can reflect the degree of genetic variation in a population. In the study, among which the highest marker of PIC is 87–30, the maximum PIC mark is 0.6786 and the minimum PIC mark is 0.2577, and the average PIC is 0.4487. Nei’s gene diversity index ranged from 0.2577 to 0.6787 with an average of 0.4487 (Table 4).

**Table 4:** Population specific summary statistics inferred from 17 SSR in populations of *A*. *gallica*.

Locus	Sample Size	Na*	Ne*	I*	Obs_Het	Exp_Het*	Nei**	Ave_Het	PIC
6–8	28	3	2.5128	0.9923	0.0000	0.6243	0.6020	0.6020	0.6020
7–11	28	3	1.6681	0.7119	0.3571	0.4153	0.4005	0.4005	0.4005
12–1	28	3	2.0524	0.8699	0.4286	0.5317	0.5128	0.5128	0.5128
10–31	28	2	1.9122	0.6700	0.0714	0.4947	0.4770	0.4770	0.4770
87–30	28	5	3.1111	1.2736	0.5000	0.7037	0.6786	0.6786	0.6786
1–12	28	3	2.0103	0.7897	0.2143	0.5212	0.5026	0.5026	0.5026
1–45	28	4	2.2659	1.0265	0.5000	0.5794	0.5587	0.5587	0.5587
1–48	28	2	1.4152	0.4692	0.3571	0.3042	0.2934	0.2934	0.2934
1–154	28	4	1.3471	0.5586	0.2857	0.2672	0.2577	0.2577	0.2577
2–5	28	2	1.5077	0.5196	0.4286	0.3492	0.3367	0.3367	0.3367
4–44	28	3	2.1189	0.8165	0.2857	0.5476	0.5281	0.5281	0.5281
4–108	28	3	1.4359	0.5586	0.3571	0.3148	0.3036	0.3036	0.3036
15–37	28	4	1.9898	0.9381	0.6429	0.5159	0.4974	0.4974	0.4974
18–10	28	4	2.4348	1.0633	0.6429	0.6111	0.5893	0.5893	0.5893
18–12	28	2	1.4152	0.4692	0.3571	0.3042	0.2934	0.2934	0.2934
20–14	28	2	1.5077	0.5196	0.4286	0.3492	0.3367	0.3367	0.3367
28–39	28	2	1.8491	0.6518	0.7143	0.4762	0.4592	0.4592	0.4592
Mean	28	3	1.9149	0.7587	0.3866	0.4653	0.4487	0.4487	0.4487
St. Dev		0.9354	0.4812	0.2393	0.1891	0.1317	0.1270	0.1270	0.1270

**Fig 4:**
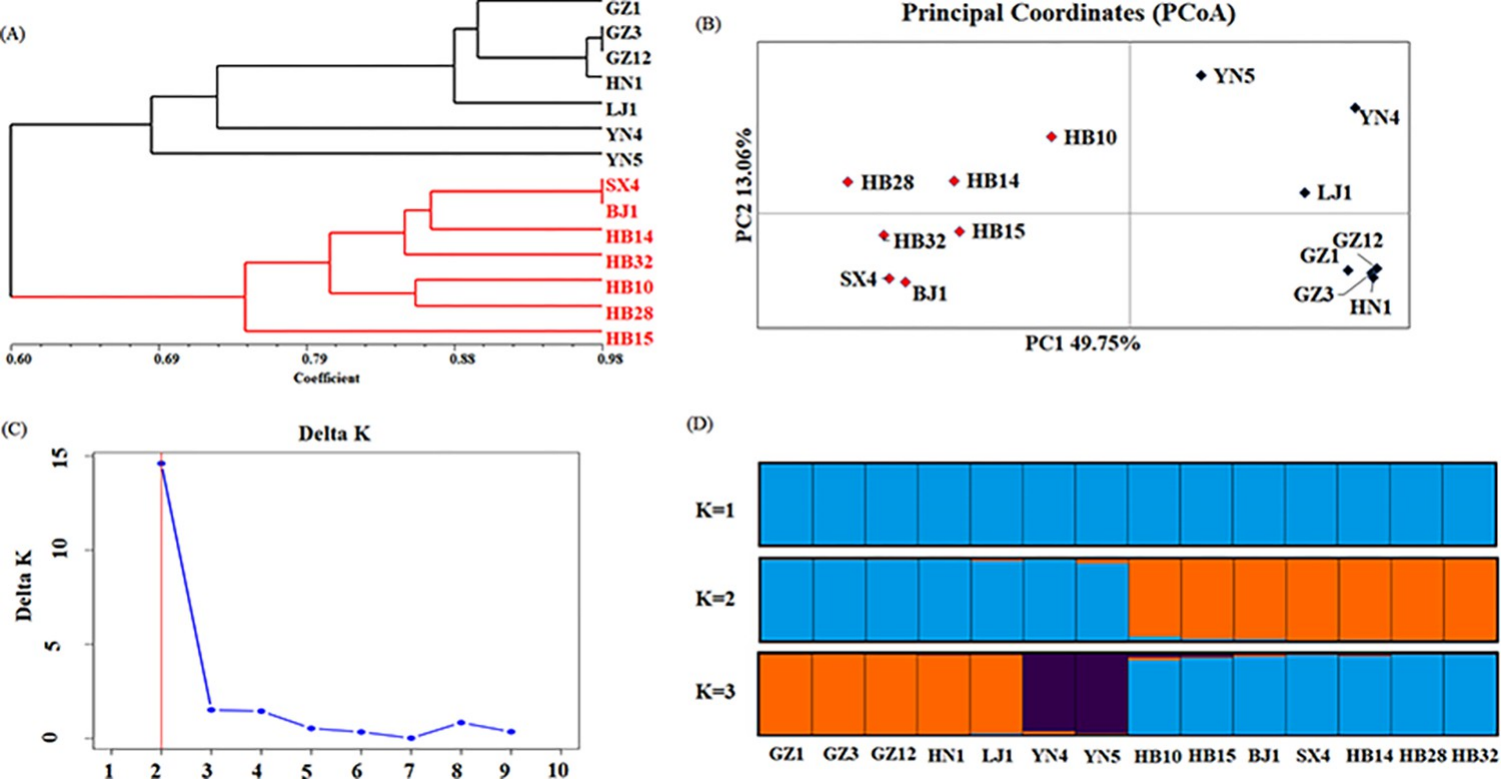
The genetic relationships of the strains of *A*. *gallica*. (A) UPGMA dendrogram demonstrating genetic relationships among the strains of *A*. *gallica*. (B) The Principal Coordinates analysis of the strains of *A*. *gallica*. (C) Two-dimensional scatter point map of *A*. *gallica* based on population genetic distance. The line graph shows the K value and △K. (D) Genetic structure of the strains of *A*. *gallica* as inferred by STRUCTURE based on 17 SSRs.

The UPGMA analysis revealed that the 14 strains of *A*. *gallica* grouped into two clusters were identified. The maximum cluster I consisted groups I, with 7 strains genotypes, respectively. The cluster II contained 7 strains were collected from Hubei and Shanxi Province (Fig 4A), which were in low-altitude *G*. *elata* producing areas mainly planted with *G*. *elata* Bl. f. elata. While Cluster I included all the strains from Guizhou and Yunnan in high-altitude *G*. *elata* producing areas mainly planted with *G*. *elata* Bl. f. glauca. The results suggested that the genetic diversity of *A*. *gallica* was subject to environmental selection including altitude and the type of *G*. *elata*.

The PCoA showed that the *A*. *gallica* strains could be divided into two subgroups (Fig 4B). PC1 was 49.75% and PC2 was 13.06%. The two subgroups were the same as those in the two clusters identified in the UPGMA analysis. The K and △K value revealed that this population could be divided into two subgroups when the K = 2 (Fig 4C). The genetic structure indicated that most of the strains originated from a single primitive ancestor, due to a few mixed individuals in each subgroup. Two subgroups could be divided with K = 2, which was highly consistent with the UPGMA analysis (Fig 4D).

## 4. Discussion

The genus Armillaria is known for its high species diversity and significant variability among its members [46]. SSRs may play a role in regulating the rapid adaptation of organisms to new environments, and the variation produced by SSRs may also be the source of rapid protein function evolution [47]. Research indicates that there are notable differences in the relative abundance and density of SSRs among different Armillaria species. Higher relative abundance and density of SSRs were observed in the genomic of the pathogenic *A*. *cepistipes* when compared to *A*. *gallica*, which grow symbiotically with *G*. *elata* and have low pathogenicity (Table 1). This observation is consistent with findings from studies on *Aspergillus* species, which SSR variability has also been observed in the pathogenic *Aspergillus* [48]. In addition, SSR quantity and density of exon region and exon region in *A*. *gallica* were opposite to those of the other two Armillaria genomes (Table 3). The results suggest that pathogenic fungus may have more SSR variability to adapt to the environment and SSRs could be a valuable marker for understanding the virulence of different Armillaria strains.

Previous studies have shown that repeat sequences are common in genic and intergenic region. SSR quantity in intergenic region was much higher than genic region. SSR density in genic region was much higher than intergenic region, with abundant trinucleotide motifs in maize [24]. In *Puccinia striiformis*, the SSR markers were mostly abundant in intergenic region [49]. In our research, we found that the SSR quantity was higher in the intergenic region than in the genic region. This result was the same to the maize genomes. However, the SSR density in intergenic region was higher than genic region in three *Armillaria* genomes and *Puccinia striiformis*, which was the opposite of maize. There results suggested that fungus had some common features in SSRs formation and structure, which may be a difference between fungal and plant genomes.

Genetic variation is an important evolutionary factor when there is strong selection pressure [24]. Higher degree of SSR variation within exon regioncould more likely cause frame-shift or mutation in protein sequence [27]. Although the SSR density was low in the *A*. *gallica* genome, the SSR density in the exon region was higher than other two genomes, and it was lower in exon region, which was rich in trinucleotide motifs (Fig 2). The variation of trinucleotide motifs in exon could cause the variation of gene function. And it may be an important factor for the evolution in *A*. *gallica* which is the most common symbiotic fungus of *G*. *elata* in China for planting or breeding research [17,50]. Therefore, genetic variation caused by genic region in *A*. *gallica* may be one of the main reasons for its evolution to symbiosis with *G*. *elata*. And the variation in the exon region may be used as the identification for *A*. *gallica*. Our analysis indicates that the proportion of polymorphic SSRs in the genic region was high than intergenic region. This may be related to the high density of SSR variants in genic region of *A*. *gallica* genome. It provides a basis for efficient selection of genomic SSR.

*A*. *gallica* plays an important role in the production of *G*. *elata*, with which it establishes a symbiotic relationship [7]. Previous studies have found that the different biological characteristics of *A*. *gallica* resulted in great differences in the growth of *G*. *elata* [51,52]. The breeding of high-quality *A*. *gallica* has played an important role in the production of *G*. *elata*. Collecting *Armillaria* species resources, analyzing the population genetic structure, and establishing a method for the identification is therefore necessary for the identification and breeding of high-quality *A*. *gallica*.

SSR is an important DNA marker in plant breeding, which could be widely used in molecular breeding and variety identification [53,54]. Previous study confirmed that multi-allelic SSRs have a higher discriminatory power than bi-allelic SNP markers to detect structure in populations at a small spatial scale with a systematic and continuous sampling design [32,55]. Sixteen markers in genome-wide SSRs were used to investigate the genetic diversity and population structure of strains in *F*. *virguliforme*, which could be grouped into three clusters [56]. In addition, three SSRs were developed to characterize the population structure of *Leptosphaeria maculans* and *L*. *biglobosa*, caused phoma stem canker (blackleg) in oilseed rape [57]. SSRs can effectively identify species. In this study, 17 SSR markers with high polymorphism were obtained, indicating that markers could be used to identify germplasm resources of *A*. *gallica*.

## 5.Conclusion

This study firstly integrated comparative genomic and genomic SSR analysis to investigate the evolution, variation and population genetic pattern of *A*. *gallica*. SSRquantity and density was higher in intergenic region than genic region. The variation of SSRs with trinucleotide motif was important for large variation in genic region of *A*. *gallica*, which provides the basis for the selection of polymorphic SSR markers. In addition, 17 high polymorphism SSRs of *A*. *gallica* genome, were successfully used for population genetic analysis of *A*. *gallica* germplasm. Overall, the variation of SSRs provide the basis for SSR markers selection in genome. SSRs will be of great value for the related genetic studies in *A*. *gallica* species. These results could be used to genetic research and identification of *A*. *gallica*, in turn promoting the production and cultivation of *G*. *elata*.

## Supporting information

S1 Table The information of *Armillaria gallica* in the experiment. (XLSX)

S2 Table Percentage of the eight types of SSR motifs in Armillaria genomes. (XLSX)

S3 Table There were 108 SSRs primers. (XLSX)

S4 Table There were 17 SSRs primers. (XLSX)

S5 Table Genetic distance and genetic correlation coefficient matrix (Below the diagonal is the genetic distance. Above the diagonal are genetic correlation coefficients). (XLSX)
